# Factors associated with adverse pregnancy outcomes of maternal syphilis in Henan, China, 2016–2022

**DOI:** 10.1017/S0950268823001589

**Published:** 2023-09-25

**Authors:** Meng Zhang, Huimin Qu, Feng Xu, Junfen Xia, Xiaoqing Hui, Hongyan Zhang, Cannan Shi, Junjian He, Yuan Cao, Mengcai Hu

**Affiliations:** Department of Health Care, The Third Affiliated Hospital of Zhengzhou University, Zhengzhou, China

**Keywords:** adverse pregnancy outcomes, associated factors, decision tree model, pregnancy, syphilis

## Abstract

Maternal syphilis not only seriously affects the quality of life of pregnant women themselves but also may cause various adverse pregnancy outcomes (APOs). This study aimed to analyse the association between the related factors and APOs in maternal syphilis. 7,030 pregnant women infected with syphilis in Henan Province between January 2016 and December 2022 were selected as participants. Information on their demographic and clinical characteristics, treatment status, and pregnancy outcomes was collected. Multivariate logistic regression models and chi-squared automatic interaction detector (CHAID) decision tree models were used to analyse the factors associated with APOs. The multivariate logistic regression results showed that the syphilis infection history (OR = 1.207, 95% CI, 1.035–1.409), the occurrence of abnormality during pregnancy (OR = 5.001, 95% CI, 4.203–5.951), not receiving standard treatment (OR = 1.370, 95% CI, 1.095–1.716), not receiving any treatment (OR = 1.313, 95% CI, 1.105–1.559), and a titre ≥1:8 at diagnosis (OR = 1.350, 95%CI, 1.079–1.690) and before delivery (OR = 1.985, 95%CI, 1.463–2.694) were risk factors. A total of six influencing factors of APOs in syphilis-infected women were screened using the CHAID decision tree model. Integrated prevention measures such as early screening, scientific eugenics assessment, and standard syphilis treatment are of great significance in reducing the incidence of APOs for pregnant women infected with syphilis.

## Introduction

Maternal syphilis can cause severe harm to pregnant women, foetuses, and infants. It not only severely affects the quality of life of pregnant women [1] but also may lead to adverse pregnancy outcomes (APOs), such as preterm birth, stillbirth, and congenital syphilis [2, 3]. Studies showed that the incidence of APOs in pregnant women infected with syphilis was significantly higher than that in pregnant women without syphilis [4, 5]. In recent years, the number of pregnant women infected with syphilis has not decreased significantly [6, 7]. It is estimated that approximately 1 million pregnant women were infected with syphilis in 2016, resulting in more than 350,000 APOs [8]. The reported incidence of syphilis in China increased from 4.50 per 100,000 in 2003 to 34.04 per 100,000 in 2021, ranking third in the national notifiable infectious diseases [9]. The number of maternal syphilis in China has also increased with the increase in the incidence of syphilis in the general population [7].

In 2007, the World Health Organization (WHO) launched the Global Initiative to Eliminate Mother-to-child Transmission of Syphilis and HIV (EMTCT) [10]. Thirteen countries have been certified by the WHO to eliminate AIDS and syphilis by 2019 [11]. In 2011, China established the integrated programme for Prevention of Mother-to-child Transmission (PMTCT) of HIV, syphilis, and HBV [12]. It aimed to prevent mother-to-child transmission by providing free testing, counselling, and treatment services for syphilis, AIDS, and hepatitis B to pregnant women [13].

Although syphilis can be completely cured with treatment, there does not appear to be a significant decrease in the incidence of APOs due to syphilis infection [14]. Several studies suggested that maternal non-standardised treatment, elevated non-treponema titre (≥1:8), and the mother in the early stage of syphilis were risk factors for congenital syphilis [14, 15]. However, few studies used the APO as an outcome indicator to assess its influence, especially based on data from pregnant women with syphilis in China.

The PMTCT Management Information System established in China can comprehensively and vertically collect data related to positive pregnant women [13, 16]. Medical and health institutions at all levels are responsible for data registration, follow-up, and investigation of case information of syphilis-infected pregnant women who come to receive medical services and for collecting basic information on syphilis-infected pregnant women, pregnancy-related conditions, syphilis-related behaviours and medical conditions, and neonatal health conditions. Henan is a populous province with the third-largest resident population in China. A large amount of maternal monitoring data was available, which provided strong data support for this study.

This study aimed to provide a scientific basis for further improving the treatment effect of syphilis infection and reducing APOs by analysing the related factors of syphilis infection and their impact on pregnancy outcomes in Henan.

## Methods

### Data source

**Figure 1.**
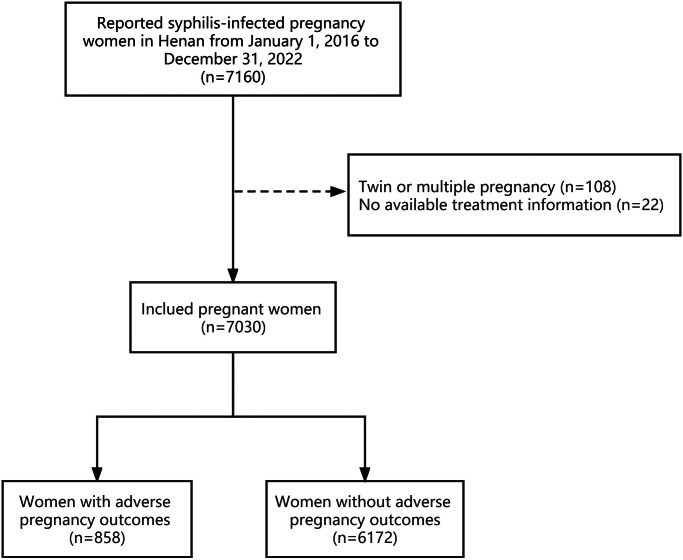
Sample size flow chart of study participants in Henan, China from 2016-2022.

Pregnant women infected with syphilis reported in Henan Province from January 2016 to December 2022 were selected as the study population, and data on demographic characteristics, treatment status, the titre at diagnosis, the titre before delivery, and APOs were extracted from the PMTCT Information System. The inclusion criteria for this study were positive serological antigen during pregnancy for both treponemal and non-treponemal, gestational age at delivery greater than or equal to 28 weeks, and singleton pregnancy. Cases of twins or multiple pregnancies or missing treatment information were excluded. Finally, 7030 cases were included (Figure 1).

### Definitions

APOs included stillbirth, death within 7 days, premature or low-birthweight infants, birth defects, neonatal pneumonia, neonatal asphyxia, and congenital syphilis. Abnormality during pregnancy mainly included premature rupture of membranes, co-infection with hepatitis B virus (HBV), and co-infection with HIV. Syphilis infection history meant that a woman has been diagnosed with syphilis before her last menstrual period of pregnancy.

We divided treatment status into three groups: standardised treatment, non-standardised treatment, and untreated. Standardised treatment for maternal syphilis was different in the 2015 and 2020 versions of the PMTCT Implementation Plan. The definition of standardised treatment was according to the 2015 version as follows: (1) application of sufficient penicillin treatment; (2) 2 courses of treatment during pregnancy, more than 2-week interval between 2 courses (benzathine benzyl penicillin treatment (240 × 104 U), bilateral hip intramuscular injection, once a week, 3 consecutive times for 1 course of treatment, or procaine penicillin treatment (80 × 104 U) per day, intramuscular injection, 15 consecutive days for 1 course); and (3) the second course of treatment is carried out and completed in the third trimester. The definition of standardised treatment is the 2020 edition as follows: (1) Complete full and sufficient penicillin treatment and (2) the treatment should be completed within 1 month before delivery. The 2015 version was implemented for positive pregnant women who gave birth between 2016 and 2020, and the 2020 version was implemented for pregnant women who gave birth between 2021 and 2022. Non-standardised treatment meant that positive women received syphilis treatment, but it was not implemented in accordance with the Implementation Plan for PMTCT (version 2015 and version 2020). Untreated referred to syphilis-infected women who had not received any treatment for syphilis.

### Statistical methods

The chi-squared test was conducted as a univariate analysis to determine whether independent variables such as demographic characteristics, clinical characteristics, syphilis treatment status, the titre at diagnosis, and the titre before delivery were independently related to APOs. We performed a multivariate analysis by considering the occurrence of APOs as independent variables and indicators with statistical significance as independent variables in the univariate analysis. Binary logistic stepwise regression was used in multivariate analysis, with the inclusion criterion α = 0.05 and exclusion criterion α = 0.10. Both univariate and multivariate analyses were performed using SPSS22.0 software. *P* values < 0.05 were considered statistically significant. The chi-squared automatic interaction detector (CHAID) is a commonly used algorithm for decision trees, which can handle nonlinear and highly collinear data that logistic regression models cannot handle, can quickly and effectively mine the main influencing factors, and display the relationship between variables well through visual results. The CHAID decision tree model was constructed using SPSS Modeler 18.0 software to screen for influences on APOs in syphilis-infected women. The classification rules are as follows: (1) The significant level of growth ‘branch’ segmentation was α_merge_ = 0.05, α_split_ = 0.05, and (2) stopping criteria were that minimum records in parent branch were less than 2% or minimum records in child branch were less than 1%.

## Results

### Participants

The average age of the 7030 pregnant women included was 28.55 ± 5.89 years, while the average age of the APO group was 28.72 ± 6.32 years. A total of 858 (12.2%) participants experienced APOs, and the proportion of APOs among syphilis-positive pregnant women in Henan Province from 2016 to 2022 ranged from 10.2% to 13.3% (Table 1).

**Table 1.** Adverse pregnancy outcomes conditions of women with syphilis infection in Henan from 2016 to 2022

Year	Total	Women with APOs		Women without APOs	
		*n*	%	*n*	%
2016	725	74	10.2	651	89.8
2017	900	117	13.0	783	87.0
2018	1,002	133	13.3	869	86.7
2019	1,088	143	13.1	945	86.9
2020	1,066	119	11.2	947	88.8
2021	1,240	149	12.0	1,091	88.0
2022	1,009	123	12.2	886	87.8

### Univariate analysis of factors affecting APOs in women infected with syphilis

There were statistically significant differences in APOs among different groups of age, education, occupation, marital status, residential area, syphilis infection history, maternal period of syphilis detection, syphilis stage, abnormality during pregnancy, and treatment status (*P* < 0.05) (Table 2). The incidence rate of APOs was the lowest in the 25- to 30-year group (11.1%) and the highest in the ≥35-year group (15.0%). The incidence rate of primary school education or lower group (15.5%) was higher than that of junior high school education or above group. The farmer group’s incidence rate (9.6%) was lower than that of the other groups. The divorced or widowed group had the highest incidence (19.6%), while the first marriage group had the lowest incidence (11.4%). The incidence rate in the urban group (13.7%) was higher than that in the rural group (11.2%). The incidence in the postpartum syphilis detection group was the highest (18.9%). The incidence in the latent syphilis group (11.7%) was lower than that in other groups. The occurring abnormality during pregnancy group (34.0%) had a higher incidence rate than the non-occurring group (9.4%). The non-standardised treatment group (13.1%) and untreated group (13.9%) had higher incidence rates than the standardised treatment group (9.8%). The group of the titre at diagnosis ≥1:8 (18.7%) had the highest incidence rate. The group of the titre before delivery ≥1:8 (22.2%) had a higher incidence rate than the negative group (9.7%), <1:8 group (10.8%), and untested group (12.9%).

**Table 2.** Participant characteristics and comparison between syphilis-infected pregnant women with and without adverse pregnancy outcomes (*n* = 7,030)

Characteristic	Total	APOs *n*(%)	Non-APOs *n*(%)	χ^2^	*P*
Age, years				13.076	0.004
___<25	1,850	237 (12.8)	1,613 (87.2)		
___25~	2,243	248 (11.1)	1,995 (88.9)		
___30~	1,833	207 (11.3)	1,626 (88.7)		
___≥35	1,104	166 (15.0)	938 (85.0)		
Education				12.360	0.006
___Primary school or lower	697	108 (15.5)	589 (84.5)		
___Junior or senior middle school	5,384	621 (11.5)	4,763 (88.5)		
___College or above	721	93 (12.9)	628 (87.1)		
___Unknown	228	36 (15.8)	192 (84.2)		
Occupation				23.269	0.000
___Farmer	3,526	338 (9.6)	3,188 (90.4)		
___Housekeeping or unemployment	2,224	296 (13.3)	1,928 (86.7)		
___Others	1,280	174 (13.6)	1,106 (86.4)		
Marital status				23.817	0.000
___Single	353	64 (18.1)	289 (81.9)		
___First married	5,527	630 (11.4)	4,897 (88.6)		
___Remarried	881	116 (13.2)	765 (86.8)		
___Cohabiting	223	39 (17.5)	184 (82.5)		
___Divorced/widowed	46	9 (19.6)	37 (80.4)		
Gravidity				6.126	0.106
___1	2,056	253 (12.3)	1,803 (87.7)		
___2	2,190	278 (12.7)	1,912 (87.3)		
___3	1,434	154 (10.7)	1,280 (89.3)		
___>3	1,350	137 (10.1)	1,213 (89.9)		
Parity				0.936	0.817
___0	2,476	309 (12.5)	2,167 (87.5)		
___1	2,760	337 (12.2)	2,423 (87.8)		
___2	1,343	163 (12.1)	1,180 (87.9)		
___>2	451	49 (10.9)	402 (89.1)		
Previous APOs				0.414	0.520
___No	5,140	619 (12.0)	4,521 (88.0)		
___Yes	1,890	239 (12.6)	1,651 (87.4)		
Residential area				9.807	0.002
___Urban	2,902	397 (13.7)	2,505 (86.3)		
___Rural	4,128	461 (11.2)	3,667 (88.8)		
Syphilis infection history				19.017	0.000
___No	3,673	388 (10.6)	3,285 (89.4)		
___Yes	3,357	470 (14.0)	2,887 (86.0)		
Maternal period of syphilis detection				14.800	0.022
___Pre-pregnancy	26	4 (15.4)	22 (84.6)		
___First trimester	1,331	155 (11.6)	1,176 (88.4)		
___Second trimester	2,225	250 (11.2)	1,975 (88.8)		
___Third trimester	2,037	260 (12.8)	1,777 (87.2)		
___At delivery	1,113	135 (12.1)	978 (87.9)		
___Postpartum	275	52 (18.9)	223 (81.1)		
___Unknown	17	2 (11.8)	15 (88.2)		
Syphilis stage				6.475	0.039
___Latent	5,500	643 (11.7)	4,857 (88.3)		
___Stages I-III	407	60 (14.7)	347 (85.3)		
___Unknown	1,123	155 (13.8)	968 (86.2)		
Abnormality during pregnancy				935.400	0.000
___Not occurred	6,239	589 (9.44)	5,650 (90.6)		
___Occurred	791	269 (34.01)	522 (66.0)		
Syphilis treatment status				19.273	0.000
___Standardized treatment	2,498	245 (9.8)	2,253 (90.2)		
___Non-standardized treatment	3,337	438 (13.1)	2,899 (86.9)		
___Untreated	1,195	166 (13.9)	1,029 (86.1)		
Titre at diagnosis				72.044	0.000
___<1:8	5,477	575 (10.5)	4,902 (89.5)		
___≥1:8	1,469	274 (18.7)	1,195 (81.3)		
Unknown	84	9 (10.7)	75 (89.3)		
Titre before delivery				94.520	0.000
___Negative	1,139	110 (9.7)	1,029 (90.3)		
___<1:8	4,454	483 (10.8)	3,971 (89.2)		
___≥1:8	862	191 (22.2)	671 (77.8)		
___Untested	575	74 (12.9)	501 (87.1)		

### Logistic regression analysis of factors affecting APOs in women infected with syphilis

Logistic regression analysis revealed that marital status, residential area, abnormality during pregnancy, syphilis infection history, treatment status, the titre at diagnosis, and the titre before delivery were associated with APOs (Table 3). First married women were less likely to have APOs than single women, with an OR of 0.657 (95% CI, 0.487–0.885). Women living in rural areas were less likely to have APOs than those in urban areas, with an OR of 0.805 (95% CI, 0.693–0.936). Compared with women without abnormality occurrence during pregnancy, women with abnormality occurrence had a higher risk of APOs (OR = 5.001, 95% CI, 4.203–5.951). Women with a syphilis infection history were more likely to have APOs than those without a syphilis infection history (OR = 1.207, 95% CI, 1.035–1.409). Women with non-standardised treatment (OR = 1.370, 95% CI, 1.095–1.716) or untreated (OR = 1.313, 95% CI, 1.105–1.559) had a greater risk of APOs than those with standardised treatment. Those with titres ≥1:8 at diagnosis had a higher risk than those with titres < 1:8, with an OR of 1.350 (95% CI, 1.079–1.690). Women with titres ≥1:8 before delivery were more likely to have APOs (OR = 1.985, 95% CI, 1.463–1.699) than negative women.

**Table 3.** Multivariate analysis of syphilis-infected pregnant women’s characteristics and APOs (*n* = 7,030)

Characteristic	OR	95% CI	*P*
Marital status			
___Single	Reference		
___First married	0.657	(0.487, 0.885)	0.006
___Remarried	0.805	(0.567, 1.144)	0.226
___Cohabiting	0.912	(0.574, 1.449)	0.696
___Divorced/widowed	0.966	(0.423, 2.208)	0.953
Residential area			
___Urban	Reference		
___Rural	0.805	(0.693, 0.936)	0.005
Abnormality during pregnancy			
___Not occurred	Reference		
___Occurred	5.001	(4.203, 5.951)	0.000
Syphilis infection history			
___No	Reference		
___Yes	1.207	(1.035, 1.409)	0.017
Syphilis treatment status			
___Standardized treatment	Reference		
___Non-standardized treatment	1.370	(1.095, 1.716)	0.006
___Untreated	1.313	(1.105, 1.559)	0.002
Titre at diagnosis			
___<1:8	Reference		
___≥1:8	1.350	(1.079, 1.690)	0.009
Unknown	0.853	(0.408, 1.783)	0.673
Titre before delivery			
___Negative	Reference		
___<1:8	1.123	(0.895, 1.409)	0.315
___≥1:8	1.985	(1.463, 2.694)	0.000
___Untested	1.213	(0.866, 1.699)	0.261

### CHAID decision tree model analysis of factors affecting APOs in women infected with syphilis

**Figure 2.**
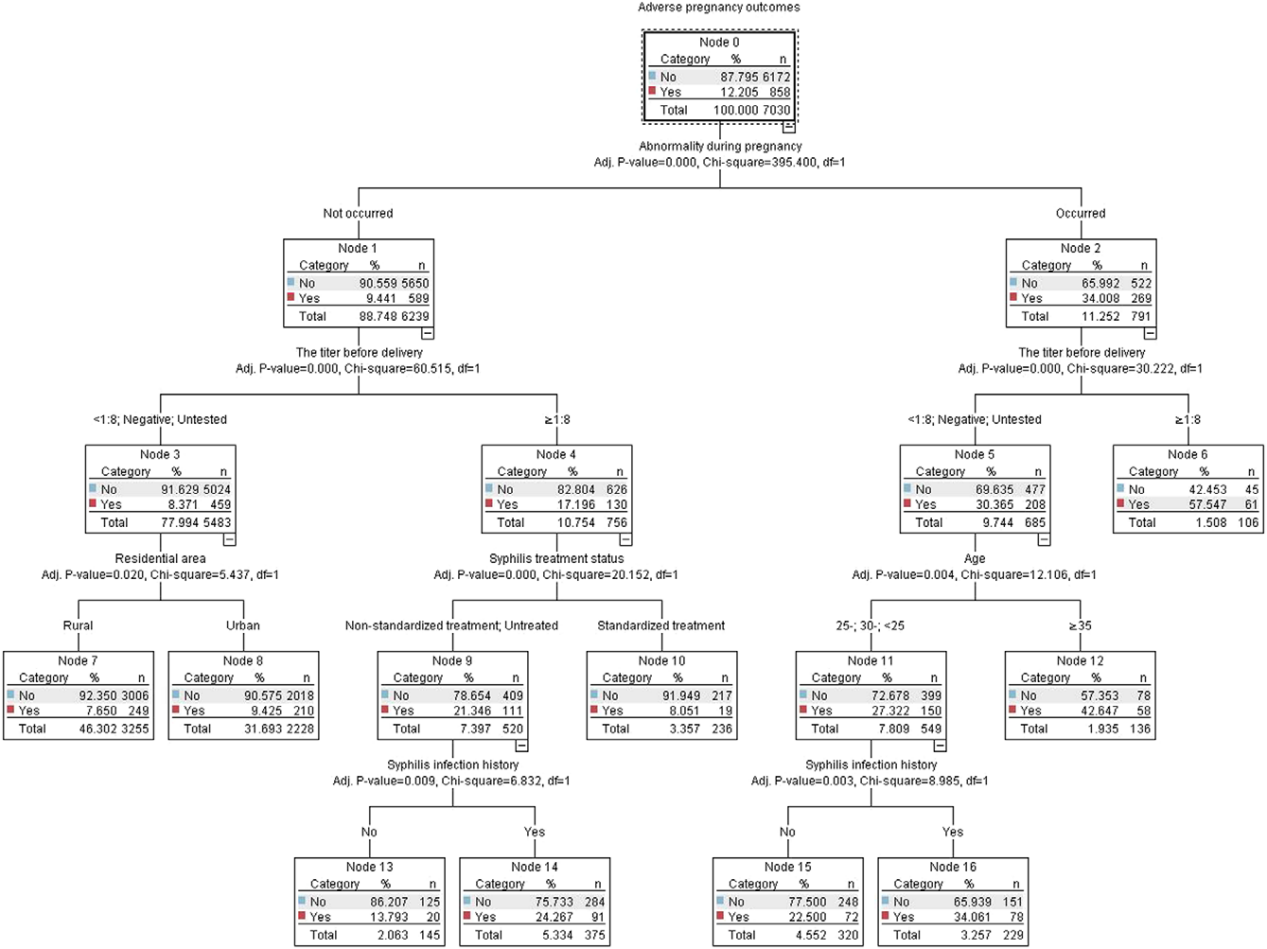
Classification tree chart of decision tree analysis on factors influencing adverse pregnancy outcomes in syphilis-infected women.

The analysis result of the CHAID decision tree analysis showed that there were 16 nodes in total and 9 terminal nodes (nodes 6, 7, 8, 10, 12, 13, 14, 15, and 16), as shown in Figure 2. The CHAID model results showed that the occurrence of abnormality during pregnancy, titres ≥1:8, living in urban areas, non-standardised treatment/untreated, age ≥ 35 years, and syphilis infection history were risk factors of APOs. The first layer of the tree was divided according to whether abnormality occurred during pregnancy, and the incidence of APOs in women with abnormality occurrence (34.0%) was significantly higher than that in women without abnormality occurrence (8.4%). The CHAID model showed that there was an interaction between abnormality during pregnancy and the titre before delivery, and the incidence of APOs in syphilis-infected women with abnormality occurrence during pregnancy and the titre ≥1:8 increased from 34.0% to 57.5%. In women with abnormalities during pregnancy, there was an interaction between the titre before delivery and age, with the incidence of APOs rising from 30.45% to 42.6% in women who were negative, not tested or had less than 1:8 titres and were 35 years or older. Among women without abnormality during pregnancy, there was an interaction between the titre before delivery and syphilis treatment status, and the incidence increased from 17.2% to 21.3% in women with the titre ≥1:8 and receiving non-standardised treatment or not receiving any treatment.

## Discussion

We conducted a retrospective cohort study and evaluated associations among the sociological characteristics, clinical features, treatment status of syphilis-positive pregnant women, and APOs. The average percentage of syphilis-positive pregnant women with APOs in Henan Province was 12.14% during the period from 2016 to 2022, and the incidence of APOs showed a relatively stable trend with no significant decline. The results of the multivariate analysis showed that marital status, history of syphilis infection, abnormality during pregnancy, syphilis treatment status, the titre before delivery, and the titre at diagnosis were associated with APOs. A total of six influencing factors of APOs in syphilis-infected women were screened using the CHAID decision tree model. Abnormality during pregnancy was the most important influence factor, followed by the titre before delivery, treatment status, age, residential area, and syphilis infection history. There was an interaction between the titre before delivery and age, and between the titre before delivery and syphilis treatment status.

The pregnant women infected with syphilis can lead to APOs such as abortion, preterm birth, and congenital syphilis. Studying the relevant factors of APOs in pregnant patients with syphilis had vital clinical value for guiding eugenics and fertility in clinical practice [17]. Overall, 57.98% of the 7030 pregnant women included in this study were reproductive active period (25–35 years). The majority of the pregnant women included has education levels of junior high school or below, and their occupations were mainly farmers, which was consistent with the characteristic distribution of the syphilis population in China [18, 19]. It was suggested that health education on syphilis prevention and control should focus on women in the reproductive active period and women of childbearing age in rural areas.

The incidence of APOs among pregnant women with syphilis in Henan from 2016 to 2022 ranged from 10.21% to 13.27%. It was reported that the incidence of APOs among syphilis-positive pregnant women in Guangzhou was 21.2% from 2014 to 2016 [20]. A study in Guangxi showed that the incidence of APOs in syphilis-positive pregnant women was 12.63% [21]. Another study conducted in Shanghai from 2001 to 2015 revealed that the average incidence of APOs was 16.72% [22]. A study in the United States showed that the premature birth rate of pregnant women infected with syphilis was 13.3% from 2016 to 2019 [23], while a study in South Korea showed no significant downward trend in the incidence of congenital syphilis from 2013 to 2018 [24]. The above findings all suggest that how to avoid APOs in syphilis-positive pregnant women remains an essential topic of research.

The results of this study suggest that women with a syphilis infection history were more likely to have APOs. Previous studies have shown that syphilis can transmit from the mother to the foetus at any stage of pregnancy, and the rate of APOs is significantly increased in foetus exposed to intrauterine syphilis, and earlier exposure to intrauterine syphilis may increase the probability of APOs [25]. Therefore, it was recommended that pregnant women with a syphilis infection history be treated for syphilis and evaluated before pregnancy. In addition, the results suggested that premarital and preconception syphilis screening is essential to prevent APOs caused by syphilis.

The results of the CHAID decision tree model showed that abnormality during pregnancy was one of the main influences on APOs. In this study, abnormalities during pregnancy mainly included premature rupture of the membranes and co-infection with HIV/HBV. Among them, premature rupture of membranes is the most common complication in the perinatal period, which can lead to an increase in the premature birth rate and perinatal mortality [26, 27]. Previous studies have shown that co-infection with HBV/HIV was a risk factor for APOs [28, 29].

Our findings suggested that APOs were less likely in women who had received standardised treatment and had low titres before delivery or at diagnosis. It has been established that penicillin treatment of maternal syphilis can reduce the incidence of APOs and avoid 98% of congenital syphilis by reducing titer or negative conversion [25]. The incidence of APOs was lower in women with standardised treatment than in those with non-standardised treatment. In addition, in Tridapalli et al. [30] study, the risk of congenital syphilis was 15 times greater when the mother received non-standardised treatment than when she received standardised treatment, which was consistent with our findings. Our study also found that syphilis-positive pregnant women with no treatment or non-standardised treatment had a higher probability of APOs than those with standardised treatment.

The standardised treatment rate in Henan Province from 2016 to 2022 was less than 50%, similar to studies in other regions of China, but lower than that in developed countries [24, 31, 32]. In China, the *Treponema pallidum* serological test is used for initial screening of pregnant women undergoing their first prenatal care. Those with positive results in the initial screening will be retested by a non-*T. pallidum* serological test while undergoing quantitative testing. It can take longer to go from a positive initial screening to a confirmed retest, rather than the same day in some medically underserved areas. This might lead to the loss of positive pregnant women and failure to receive timely treatment [33, 34]. This also reminded us that optimising the maternal syphilis screening process was essential for increasing the standardised treatment rate for positive pregnant women. In addition, it was our understanding that some positive pregnant women would have been treated at sexually transmitted infection (STI) clinics and that the standard of treatment there was not consistent with the standard in the technical aspects of PMTCT intervention services, resulting in some positive pregnant women not receiving standardised treatment. It was essential to improve the standardisation of treatment rates, to standardise the diagnosis and treatment process of maternal syphilis, and to conduct training on key technical points for PMTCT covering all departments involved. It is also necessary to explore and evaluate the optimal and most effective management models for maternal syphilis.

Our study, using province-wide cumulative data, is the first to analyse the influencing factors of APOs among syphilis-positive pregnant women in Henan Province, providing a basis for formulating implementation plans to PMTCT of syphilis. This study also had several limitations. First, the data used in this study were monitoring data from the information system. Although the Henan provincial administration conducted quarterly quality control and supervision of the data reported by institutions, it was found that a small number of institutions in some areas had missing or misreported data, which might lead to a bias in the results. Second, this study did not examine other risk factors of APOs that were not adequately collected in the information systems. In addition, this study did not include pregnant women with syphilis who were delivered at less than 28 weeks, which may have underestimated the incidence of APOs.

## Conclusion

There are many factors that affect APOs in syphilis-infected women, requiring comprehensive multi-sectoral prevention and treatment efforts. Screening for syphilis before marriage and pregnancy, standardised eugenics guidance, and evaluation and standardised treatment for syphilis are of great significance in reducing the incidence of APOs such as premature birth, stillbirth, birth defects, and congenital syphilis. It is necessary to strengthen health education related to PMTCT for syphilis-positive pregnant women to improve their treatment compliance. Maternity and perinatal care institutions and relevant management departments should further standardise maternal risk management for syphilis-positive pregnant women, improve standard treatment rates, and reduce the incidence of APOs.

## Data Availability

Data are available upon request from the corresponding author.

## Abbreviations

AIDS: acquired immune deficiency syndrome
APOs: adverse pregnancy outcomes
CHAID: chi-squared automatic interaction detector
EMTCT: eliminate mother-to-child transmission of syphilis and HIV
PMTCT: prevention of mother-to-child transmission
STIs: sexually transmitted infections
WHO: World Health Organization
